# Caries Preventive Interventions and Oral Health Inequalities: A Scoping Review

**DOI:** 10.1177/23800844221109116

**Published:** 2022-07-31

**Authors:** A.W. van Meijeren-van Lunteren, Y. You, H. Raat, E.B. Wolvius, L. Kragt

**Affiliations:** 1 The Generation R Study Group, Erasmus University Medical Centre, CA Rotterdam, the Netherlands; 2 Department of Oral & Maxillofacial Surgery, Special Dental Care and Orthodontics, Erasmus University Medical Centre, CA Rotterdam, the Netherlands; 3 Department of Public Health, Erasmus University Medical Centre, CA Rotterdam, the Netherlands

**Keywords:** health disparities, public health, social determinants, dental health, health promotion, community dentistry

## Abstract

**Introduction:**

Dental caries remains one of the most prevalent but preventable diseases among children worldwide and especially affects children with a lower socioeconomic status or ethnic minority background. It is important that all groups of children are reached by preventive interventions to reduce oral health inequalities. So far, it is unknown whether children from different social and ethnic groups benefit equally from potentially effective oral health interventions.

**Objectives:**

This scoping review aimed to identify European public health interventions that report their effect on dental caries across different social groups.

**Methods:**

Four databases were searched for studies evaluating the effect of oral health interventions on dental caries among children from 0 to 12 y, and studies were included when results were presented by children of different social groups separately.

**Results:**

A total of 14 studies were included, representing 4 different countries: 3 randomized and 11 nonrandomized studies. Most studies were performed at schools. Six studies showed results indicative of a reduction in oral health inequalities, 4 studies showed results that potentially widen oral health inequalities, and 5 studies showed results that were indicative of no impact on oral health inequalities. Interventions that contain early approaches, with a high frequency, approaching multiple levels of influence, and including at least the broader organizational or public policy level, may have the potential to reduce oral health inequalities among children from birth to young adolescence.

**Conclusion:**

We recommend researchers to perform high-quality intervention studies and to evaluate the effectiveness of oral health intervention always in different socioeconomic or ethnic groups separately, to better understand their contribution toward oral health (in)equalities.

**Knowledge Transfer Statement:**

This review offers insight in the differential effects that oral health interventions might have across different social groups. Its results can be used to develop interventions that might reduce oral health inequalities among children. Also, we recommend future researchers to always evaluate the effects of any preventive oral health measure in different social groups separately.

## Introduction

Dental caries is one of the most prevalent, but neglected, noncommunicable diseases among children worldwide (The Lancet 2009; Kassebaum et al. 2015). Dental caries is preventable, and it is known that preventive care has positive long-term consequences in terms of well-being and cost-effectiveness (Selwitz et al. 2007; The Lancet 2009; Pitts et al. 2017). Therefore, especially intervening at a young age is important to maintain proper oral health behaviors during adulthood (Halfon and Hochstein 2002). Dental caries among children has been reduced in European countries in the previous decade, but still a stagnation or even worsening in its prevalence prevails (Watt and Sheiham 1999; Pitts et al. 2017; Schuller et al. 2018). Especially socially disadvantaged children, indicated by low socioeconomic status or ethnic minority background, are more often affected by poor oral health, including dental caries (Watt and Sheiham 1999; Schuller et al. 2018). Therefore, in a recent report from the World Health Organization (WHO), for setting goals for 2030, concerns are raised about the existing oral health inequalities that were not reduced in the past years and due to the COVID-19 pandemic might be even further increased (WHO 2021).

Several oral health interventions that could prevent dental caries among children do exist on an international basis, and previous researchers have summarized and evaluated those for their effectiveness (dos Santos et al. 2013; de Silva et al. 2016). Also, 1 systematic review has been performed on dental preventive strategies among disadvantaged children solely (Skeie and Klock 2018), and 1 recent review summarized the effect of intervention studies on oral health inequalities based on the analyses, reporting, and interpretation of the individual studies (Shen et al. 2021). However, so far, the effects of oral health interventions have not been objectively summarized and evaluated for different groups of social status separately. This is alarming since from research beyond the field of oral health, it is known that effective interventions can increase health inequalities as children belonging to affluent social groups (e.g., high socioeconomic status or ethnic majority background) are reached better and benefit more from preventive interventions than disadvantaged groups (e.g., low socioeconomic status or ethnic minority background) (Lorenc et al. 2013). So far, no study has been performed to gain more insight into potential reasons for the differential impact of interventions on oral health in a systematic manner. Insight in characteristics of interventions that could reduce or induce oral health inequalities could help future researchers and policy makers in the development of oral health intervention programs. Moreover, previous reviews include intervention studies that were carried out in the United States, Australia, Brazil, or low-income countries. Even though the oral health of inhabitants among European countries is relatively good compared to other continents, at the same time, oral health inequalities exist in all European countries (Forster et al. 2018). Especially, because most European countries pay limited attention to oral health inequalities within their policy, the need for research in oral health promotion to develop comprehensive strategies is urgent (Patel 2012). Given the limited insight in the prevention and reduction of oral health inequalities, a scoping review was performed. Scoping reviews are a type of review that is commonly used to explore and summarize evidence of all available literature, to identify knowledge gaps, and to inform future research, especially when gaining insight in intervention programs (Peters et al. 2020). Therefore, the aim of this scoping review was to identify European public health interventions that report their effect on dental caries in children across different social groups, to summarize the effect of these interventions across different social groups in a narrative manner, to determine the potential direction on oral health inequalities of each intervention study, and to identify common attributes of interventions according to their potential direction on oral health inequalities.

## Methods

### Protocol and Registration

Scoping reviews are used to identify and provide an overview of the available evidence. The main consideration to perform a scoping review is when it is not aimed to produce a synthesized answer to the research question (Munn et al. 2018). Therefore, to answer the aims of this review, a scoping review was conducted. The review reports according to the PRISMA extension for conducting Scoping Reviews (PRISMA-ScR) (Tricco et al. 2018). The review protocol was published on PROSPERO (CRD42020156635).

### Eligibility Criteria

- Population: healthy children from 0 to 12 y, without any other preexisting (medical) condition or needs
- Intervention: any public health intervention to prevent dental caries, performed at home, schools, general health services, or in a community setting, that addresses participants of all social groups (universal interventions) and the effects needed to be reported in different groups of social position separately. Social disadvantage was based on differences in social participation, cultural differences, and language barriers indicated by the following (Galobardes et al. 2007): ethnic minority background, immigrant status, children from families with lower socioeconomic status (SES) indicated by low parental educational level, low household income, living in deprived areas, or low parental occupational class. Only studies conducted in European countries were included.
- Comparator: control group or intervention (i.e., no intervention, standard preventive care, or basic intervention program)
- Outcome: dental caries in the primary or permanent dentition assessed by a trained examiner
- Study design: randomized controlled trials (RCTs) and non-RCTs, including quasi- or nonrandomized controlled studies, controlled before-and-after studies, interrupted time-series studies, and comparisons with historical controls or national trends

### Information Sources and Search

A literature search was conducted in EMBASE, Medline (Ovid), Web of Science core collection, and Cochrane Central Register of Controlled Trials in November 2019, and a search update was performed in March 2021. The full search strategy was built with the support of the librarian of the Erasmus Medical Centre and is available in the supplementary file. The search was initially designed for EMBASE and adapted for all other databases. Search terms for dental caries were combined with terms for preventive medicine, intervention studies, social groups, and children. No date and language restrictions were applied.

For the selection of gray literature and unpublished results, to limit the risk of reporting bias, we performed an electronic search in WorldCat, Scientific Electronic Library Online (scielo.org), opengrey.eu, Open Access Theses and Dissertations (oatd.org), and Google Scholar, based on the following keywords: intervention, oral health, caries prevention, public health, and socially disadvantaged children.

### Study Selection

Titles and abstracts were independently screened by 2 researchers (AM-L and YY) using EndNote to make the initial selection. All potentially relevant studies were read full-text by 2 researchers (AM-L and YY). In case of discrepancy, a third reviewer (LK) was consulted. Thereafter, reference lists of systematic reviews identified in the literature search and of all included studies were screened for additional relevant studies.

### Data Extraction

From the final set of relevant studies, the following data were extracted: study characteristics (first author, publication year, country, study design, study population size, age, duration, setting), type of intervention, control group, outcome(s), outcome assessment, the measures of social disadvantage (e.g., indicator of SES, ethnic background, or of area deprivation score), and effect size measures used.

### Analysis

The effect on dental caries of each intervention was evaluated for the lowest and highest group of social disadvantage and assessed by mean differences (MDs) or risk ratios (RRs) with corresponding confidence intervals (CIs) calculated by Student’s *t* test or the Mantel–Haenszel procedure with a significance level of *P* < 0.05. To identify the potential direction of each intervention on oral health inequalities, the (significance of) effect estimates were compared. Due to the differences in study design and outcome measures and statistical heterogeneity, no statistical synthesis of results was performed, and no pooled summary measures were presented. We categorized the differential effects of each intervention per social group as follows: 1) the intervention is indicative of a potential reduction in oral health inequalities: the intervention preferentially reduced dental caries in the most disadvantaged group; 2) the intervention is indicative of a potential widening of oral health inequalities: the intervention preferentially reduced dental caries in the least disadvantaged group; and 3) the intervention is indicative of no preferential impact on oral health inequalities: the intervention equally reduced dental caries in both social groups or did not show an overall effect. Accordingly, we reported determinants of the interventions, such as type, length, frequency, setting, or the level of influence. The level of influence on health behavior was categorized as individual (knowledge, attitudes, and beliefs), interpersonal (family, friends, peers, or interactions), organizational (institutionalized rules or regulations), community (social networks and norms), and policy level (local, regional, or national policies and laws) (McLeroy et al. 1988; Rimer and Glanz 2005).

### Risk of Bias

This is a scoping review, and generally all available evidence regardless of the methodological quality is included. In order to examine how previous research was conducted and which elements contribute to the quality of the evidence, we additionally present a risk of bias analysis. These results will give guidance and recommendation for future research. To assess the risk of bias in the individual studies, the Quality Assessment Tool for Quantitative Studies from the Effective Public Health Practice Project was used (Effective Public Healthcare Panacea Project 1998; Jackson and Waters 2005). This is a validated assessment method to rate the methodological quality of both randomized and nonrandomized intervention studies recommended for evaluating public health programs (Jackson and Waters 2005; Armijo-Olivo et al. 2012). The tool includes 6 components, including selection bias, design, confounders, blinding, data collection methods, and withdrawals and dropouts. Study components were rated as strong, moderate, or weak, and the study was rated strong if none of the components were rated weak, moderate if 1 component was rated weak, or weak if 2 or more components were rated weak.

## Results

### Selection of Studies

The study selection is summarized in the Figure. Electronic searches retrieved a total of 3,639 unique references. Titles and abstracts were screened, and 174 studies were selected for full-text screening. Finally, a selection of 14 articles was included in the present study. The main reasons for exclusion were non-European countries, no stratification into social groups, no evaluation of an intervention program, or studies without a control group.

**Figure.**
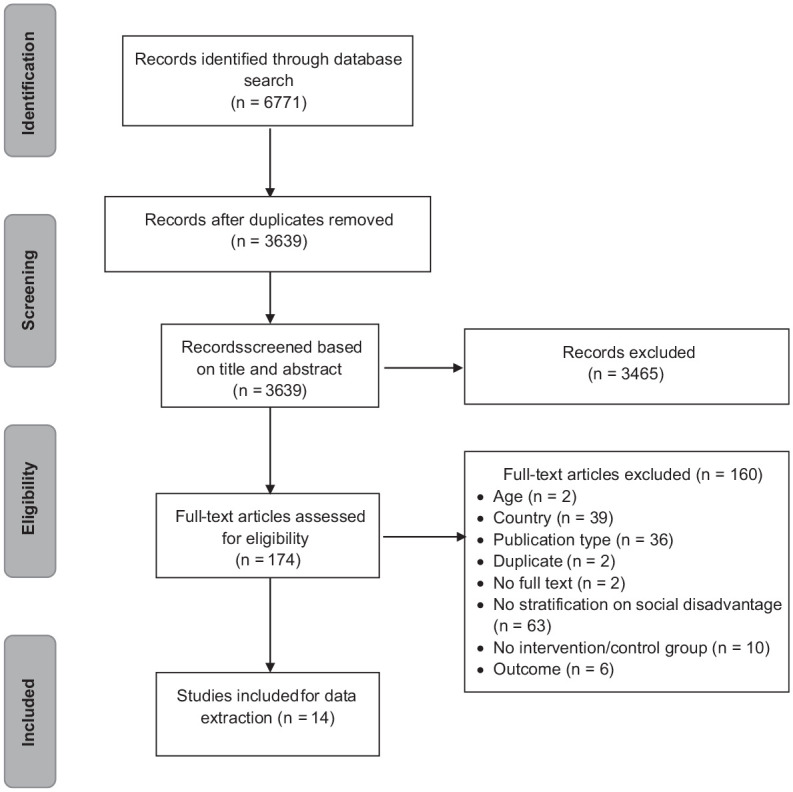
PRISMA flow diagram for study selection. Flow diagram shows the selection of studies that were retrieved, screened, and selected for inclusion.

### Included Studies

A summary of the included studies is presented in Table 1. The date of publication ranged from 1989 to 2020. Of the 14 included studies, 3 were randomized controlled trials (Ellwoodet al. 2004; Qadri et al. 2018; Winter et al. 2018), 7 were quasi-experimental or nonrandomized studies (Winter et al. 1989; Evans et al. 1996; Freeman et al. 2001; Levin et al. 2009; Drosen et al. 2010; Wagner and Heinrich-Weltzien 2017; Kidd et al. 2020), and 4 were historical comparison studies, including repeated cross-sectional surveys (Dargent-Paré et al. 1999; Heinrich-Weltzien et al. 2007; McMahon et al. 2011; MacPherson et al. 2013). Of all studies, 5 were conducted in Germany (Winter et al. 1989; Heinrich-Weltzien et al. 2007; Drosen et al. 2010; Wagner and Heinrich-Weltzien 2017; Qadri et al. 2018), 7 in the United Kingdom (Evans et al. 1996; Ellwood et al. 2004; Levin et al. 2009; McMahon et al. 2011; MacPherson et al. 2013; Winter et al. 2018; Kidd et al. 2020), 1 in France (Dargent-Paré et al. 1999), and 1 in Ireland (Freeman et al. 2001). Three studies evaluated 1 or more components of the “Childsmile” oral health program (McMahon et al. 2011; MacPherson et al. 2013; Kidd et al. 2020). Most of the included studies were performed at nursery and primary schools (Dargent-Paré et al. 1999; Freeman et al. 2001; Heinrich-Weltzien et al. 2007; Levin et al. 2009; Drosen et al. 2010; MacPherson et al. 2013; Qadri et al. 2018; Winter et al. 2018; Kidd et al. 2020), 2 were performed at home (Winter et al. 1989; Ellwood et al. 2004), 1 was performed in a community setting (Evans et al. 1996), and 2 had a mixed setting at home and at a dental clinic (McMahon et al. 2011; Wagner and Heinrich-Weltzien 2017). Seven studies assessed caries in the primary dentition (Ellwood et al. 2004; Evans et al. 1996; Kidd et al. 2020; MacPherson et al. 2013; McMahon et al. 2011; Wagner and Heinrich-Weltzien 2017; Winter et al. 1989), 5 assessed caries in the permanent dentition (Dargent-Paré et al. 1999; Freeman et al. 2001; Heinrich-Weltzien et al. 2007; Levin et al. 2009; Qadri et al. 2018), and 2 studies assessed both (Drosen et al. 2010; Winter et al. 2018). The following indicators were used to measure social disadvantage of the children: parental employment status (Winter et al. 1989; Evans et al. 1996; Dargent-Paré et al. 1999), poverty (Freeman et al. 2001), area deprivation (Ellwood et al. 2004; Levin et al. 2009; McMahon et al. 2011; MacPherson et al. 2013; Kidd et al. 2020), ethnic background (Dargent-Paré et al. 1999; Heinrich-Weltzien et al. 2007; Drosen et al. 2010), or a summary score of 2 or more socioeconomic indicators (Wagner and Heinrich-Weltzien 2017; Qadri et al. 2018; Winter et al. 2018).

**Table 1.** Characteristics of Included Intervention Studies.

Reference	Country	Study Type	Setting	Intervention Group	Comparison Group	Indicator of Social Disadvantage	Dental Outcomes	Age	Methodo-logical Quality^a^
Dargent- Paré et al. (1999)	France	Historical comparison	Primary school	Multicomponent intervention: provision fluoride tablets; authorization fluoridated salt; promotion fluoridated toothpaste; training and communication; mouthwashes and brushing with fluoride	No intervention	Ethnic background; parental employment status	Mean DMFT	11 y	Weak
Drosen et al. (2010)	Germany	Quasi experimental study	Primary school	Intensive program next to basic program: yearly preparation of healthy breakfast, brushing exercises, visualization of plaque, fluoride varnish, educational games	Basic program: yearly instruction on caries prevention, practicing dental flossing, supervised toothbrushing	Ethnic background	Mean dmft, DMFT, dmfs and DMFS	10 y	Weak
Ellwood et al. (2004)	United Kingdom	RCT	Home	Group 1: free toothpaste (1,450 ppm fluoride), toothbrush, dental health literature Group 2: free toothpaste (440 ppm fluoride), toothbrush, dental health literature	Group 3: no intervention	Area deprivation	Mean and proportion dmft	5 y	Weak
Evans et al. (1996)	United Kingdom	Quasi experimental study	Community	Children living in Newcastle where water has been fluoridated for 25 y	Children living in Northumberland where water is nonfluoridated	Paternal employment status	Mean dmft	5 y	Moderate
Freeman et al. (2001)	Ireland	Quasi-experimental study	Primary school	Consumption of only milk and fruit at break time	No intervention	Relative poverty and social deprivation	Proportion DMFT	10 y	Moderate
Heinrich- Weltzien et al. (2007)	Germany	Historical comparison	Kindergarten and primary school	Preventive program since kindergarten: twice a year brushing exercises, once a year dental health screening, nutrition events, teaching on dental health and teeth cleaning, and 3 times per year fluoride varnish	Basic dental program in primary school years: lessons on oral hygiene techniques and knowledge on caries prevention once per year	Ethnic background	Mean DMFT	12 y	Weak
Kidd et al. (2020)	United Kingdom	Quasi-experimental	Nursery school	Daily supervised toothbrushing (part of Childsmile program): Group 1: ≤1 y Group 2: >1–2 y Group 3: >2–3 y Group 4: >3 y	No intervention	Area deprivation	Obvious caries experience	5 y	Strong
Levin et al. (2009)	United Kingdom	Quasi-experimental study	Primary school	Biweekly supervised mouth rinsing	No intervention	Area deprivation	Mean and proportion D3MFT	11 y	Moderate
Mac- Pherson et al. (2013)	United Kingdom	Historical comparison	Nursery school and home	Daily supervised toothbrushing and free fluoride toothpaste (part of Childsmile program)	No intervention	Area deprivation	Mean d3mft	5 y	Weak
McMahon et al. (2011)	United Kingdom	Historical comparison	Community, dental practice, home, nursery, and primary school	Provision of toothbrush and toothpaste; daily supervised toothbrushing at nursery schools; healthy snacks and drinks in nurseries and schools (part of Childsmile program)	No intervention	Area deprivation	Mean and proportion d3mft	3 y	Weak
Qadri et al. (2018)	Germany	Cluster RCT	Primary school	Oral health program in school curricula. Several activities delivered by schoolteachers	No intervention	Social index based on educational level, vocational training, net household income, and parental employment status	Incidence rate ratio DMFT	10 y	Strong
Wagner and Heinrich- Weltzien (2017)	Germany	Quasi-experimental study	Home and dental clinic	Oral health counseling in first month after birth, comprehensive dental care by dentist, fluoride varnish, maternal counseling	Oral health counseling in first month after birth, maternal counseling	Social status based on parental education and employment status	Mean d1–4mfs, d3–4mfs	5 y	Moderate
Winter et al. (1989)	Germany	Controlled clinical trial	Home	Free toothpaste (1,055 ppm fluoride) and toothbrush	Free toothpaste (550 ppm fluoride) and toothbrush	Paternal employment status	Mean and proportion dmfs	5 y	Strong
Winter et al. (2018)	United Kingdom	RCT	Kindergarten and primary school	Group 1: daily supervised toothbrushing with fluoride toothpaste (500 ppm fluoride), 3–4 times brushing exercise, free toothbrush and toothpaste at kindergarten. Toothbrushing with fluoride gel (12,500 ppm fluoride) at primary school. Group 2: daily supervised toothbrushing with fluoride toothpaste (500 ppm fluoride), 3–4 times brushing exercise, free toothbrush and toothpaste at kindergarten. 3–4 times per year instruction on toothbrushing at primary school. Group 3: 3–4 times brushing exercise and free toothbrush and toothpaste at kindergarten. Toothbrushing with fluoride gel (12,500 ppm fluoride) at primary school. Group 4: 3–4 times brushing exercise and free toothbrush and toothpaste at kindergarten. 3–4 times per year instruction on toothbrushing at primary school. Group 5: no intervention at kindergarten. Toothbrushing with fluoride gel (12,500 ppm fluoride) at primary school.	Group 6: no intervention at kindergarten. 3–4 times per year instruction on toothbrushing at primary school.	Social index based on parental educational, income, and occupational status	Mean increment dmft, DMFT	9 y	Moderate

dmft, decayed missing filled teeth in primary dentition; DMFT, decayed missing and filled teeth in permanent dentition; dmfs, decayed missing and filled surfaces in primary dentition; DMFS, decayed missing and filled surfaces in permanent dentition; d3mft, modification of dmft that assesses caries only if the decay affects the dentin layer; D3MFT, modification of DMFT that assesses caries only if the decay affects the dentin layer; d1–4mfs, modification of dmfs that assesses caries reaching both enamel and dentin; d3–4mfs, modification of dmfs that assesses caries only if the decay affects the outer and inner dentin layers; NaF, sodium fluoride; RCT, randomized controlled trial; ppm, parts per million.

a To assess the risk of bias in the individual studies, the quality assessment tool for quantitative studies from the effective public health practice project was used.

### Quality of Included Studies

In Table 1, the global rating of the methodological quality of the included studies is presented, and an extended table including the rating of all 6 components is presented in Appendix Table 1. Of the included studies, 3 were rated as strong (Winter et al. 1989; Qadri et al. 2018; Kidd et al. 2020), 5 were rated as moderate (Evans et al. 1996; Freeman et al. 2001; Levin et al. 2009; Wagner and Heinrich-Weltzien 2017; Winter et al. 2018), and 6 were rated as weak in terms of study quality (Dargent-Paré et al. 1999; Ellwood et al. 2004; Heinrich-Weltzien et al. 2007; Drosen et al. 2010; McMahon et al. 2011; MacPherson et al. 2013).

### Effect of Interventions across Social Groups

#### Interventions that may reduce inequalities

Six studies showed results that were indicative of a reduction in dental health inequalities (Evans et al. 1996; McMahon et al. 2011; MacPherson et al. 2013; Wagner and Heinrich-Weltzien 2017; Winter et al. 2018; Kidd et al. 2020) (Table 2 and Appendix Table 2). The first study showed that children living in the fluoridated areas had statistically significantly lower caries levels than children in the nonfluoridated areas. Moreover, children in the low social class showed a higher reduction in dental caries (Evans et al. 1996). The second study showed that children from the most deprived areas had a significantly lower risk of dental caries when being involved in supervised toothbrushing for 1, 2, or 3 or more years than children who did not receive any supervised toothbrushing. Children from the least deprived areas only showed a reduced risk of dental caries when they received supervised toothbrushing for 3 y or longer, but the absolute risk reduction among the most deprived children was higher (Kidd et al. 2020). The third study evaluated a combination of daily supervised toothbrushing and provision of fluoride toothpaste, using an ecological approach. Children in the intervention group from both deprived and nondeprived groups showed a significant reduction in dental caries, but the absolute reduction in caries was highest among the most deprived group (MacPherson et al. 2013). A fourth study showed that children from both the least and most deprived areas who received the “Childsmile” intervention showed significantly less dental caries. The absolute change in dental caries was highest among children from the most deprived areas (McMahon et al. 2011). The fifth study compared an extensive oral health program (including oral health counseling, dental care, fluoride varnish) with the basic oral health program (including oral health counseling only). The intervention was effective in 5-y-old children from both socioeconomic groups, but the reduction in caries, compared to the control group, was the largest in the low SES group (Wagner and Heinrich-Weltzien 2017). The sixth study showed that children in intervention group 2 (receiving supervised toothbrushing, toothbrushing exercise, free toothbrush and toothpaste at kindergarten, and instructions on toothbrushing at primary school) and with low SES had statistically significantly lower dental caries compared to the control group, which received basic dental education at primary school only. Among children of high SES, this decrease in dental caries was not observed (Winter et al. 2018).

**Table 2.** Effectiveness of Interventions on Dental Caries Stratified per Social Group and the Potential Impact on Oral Inequalities.

Reference	Outcome	Socially Disadvantaged Group	MD (95% CI)	RR (95% CI)	Potential Impact on Oral Inequalities^a^
Dargent-Paré et al. (1999)	DMFT	Employed father	**–1.42 (–1.57, –1.27)**		↑
		Unemployed father	**–1.13 (–1.76, –0.50)**		
Drosen et al. (2010)	dmft/DMFT	Ethnic majority	0.30 (–0.48, 1.08)		*↔*
		Ethnic minority	–0.50 (–1.38, 0.38)		
Ellwood et al. (2004)	dmft	Group 1^b^			
		Least deprived	**–0.50 (–0.93, –0.07)**		↔
		Most deprived	–0.50 (–1.01, 0.01)		
		Group 2^b^			↔
		Least deprived	0.30 (–0.19, 0.79)		
		Most deprived	–0.30 (–0.86, 0.26)		
Evans et al. (1996)	dmft	High social class	**–0.87 (–1.51, –0.23)**		↓
		Low social class	**–1.57 (–2.93, –0.21)**		
Freeman et al. (2001)	DMFT	High SES		1.30 (1.00, 1.70)	↔
		Low SES		1.02 (0.85, 1.23)	
Heinrich-Weltzien et al. (2007)	DMFT	German students at Grammar schools	**–1.50 (–1.87, –1.13)**		↑
		German students at secondary modern schools	**–0.70 (–1.33, –0.07)**		
		Turkish students at secondary modern schools	–0.50 (–1.11, 0.11)		
Kidd et al. (2020)	Caries experience observed	Group 1^b^ Least deprived Most deprived		1.20 (0.97, 1.48)**0.87 (0.80, 0.96)**	↓
		Group 2^b^			
		Least deprived		1.07 (0.92, 1.25)	↓
		Most deprived		**0.86 (0.79, 0.93)**	
		Group 3^b^			
		Least deprived		1.04 (0.90, 1.19)	↓
		Most deprived		**0.86 (0.80, 0.93)**	
		Group 4^b^			
		Least deprived		**0.80 (0.66, 0.96)**	↓
		Most deprived		**0.73 (0.65, 0.81)**	
Levin et al. (2009)	D3MFT	Least deprived	**–0.50 (–0.93, –0.07)**		↑
		Most deprived	–0.31 (–1.47, 0.85)		
MacPherson et al. (2013)	d3mft	Least deprived	**–0.43 (–0.60, –0.25)**		↓
		Most deprived	**–1.71 (–1.93, –1.49)**		
McMahon et al. (2011)	d3mft	Least deprived	**–0.20 (–0.34. –0.06)**		↓
		Most deprived	**–1.00 (–1.24, –0.76)**		
Qadri et al. (2018)	∆DMFT	High SES		**0.09**	↑
		Low SES		1.43	
Wagner and Heinrich-Weltzien (2017)	d1–4mfs	High SES	**–2.60 (–4.61, –0.59)**		**↓**
		Low SES	**–11.50 (–18.62, –4.38)**		
Winter et al. (1989)	dmfs	Nonmanual occupation class	–0.05 (–0.90, 0.80)		*↔*
		Manual occupation class	0.60 (–0.32, 1.52)		
Winter et al. (2018)	∆dmft/DMFT	Group 1^b^	Group 6^b^		
		High SES	–0.25 (–0.72, 0.22)		*↔*
		Low SES	–0.10 (–0.63, 0.43)		
		Group 2^b^	Group 6^b^		
		High SES	0.09 (–0.65, 0.83)		**↓**
		Low SES	** –0.38 (–0.75, –0.01)**		
		Group 3^b^	Group 6^b^		
		High SES	–0.08 (–0.45, 0.29)		*↔*
		Low SES	0.28 (–0.15, 0.71)		
		Group 4^b^	Group 6^b^		
		High SES	–0.23 (–0.61, 0.15)		*↔*
		Low SES	0.37 (–0.05, 0.79)		
		Group 5^b^	Group 6^b^		
		High SES	–0.04 (–0.42, 0.34)		*↔*
		Low SES	0.01 (–0.27, 0.29)		

An extended table is in the supplementary file (Appendix Table 2). Bold font effect estimates indicate statistical significance (*P* < 0.05).

CI, confidence interval; dmft, decayed missing filled teeth in primary dentition; DMFT, decayed missing and filled teeth in permanent dentition; dmfs, decayed missing and filled surfaces in primary dentition; DMFS, decayed missing and filled surfaces in permanent dentition; d3mft, modification of dmft that assesses caries only if the decay affects the dentin layer; D3MFT, modification of DMFT that assesses caries only if the decay affects the dentin layer; d1–4mfs, modification of dmfs that assesses caries reaching both enamel and dentin; d3–4mfs, modification of dmfs that assesses caries only if the decay affects the outer and inner dentin layers; MD, mean difference; RR, risk ratio; SES, socioeconomic status; ∆, increment.

a The potential impact on oral health inequalities is reported using symbols indicating the following: ↓ if the intervention is likely to reduce inequalities; ↑ if the intervention is likely to widen inequalities; and ↔ if the intervention had no differential impact on the 2 groups.

b The groups refer to different intervention elements as indicated in Table 1.

#### Interventions that may widen inequalities

Four studies showed results that were indicative of a widening in oral health inequalities (Dargent-Paré et al. 1999; Heinrich-Weltzien et al. 2007; Levinet al. 2009; Qadri et al. 2018) (Table 2 and Appendix Table 2). One study showed that children from both employed and unemployed fathers who received an intervention program, including several elements (including provision and promotion of several fluoride measures), showed significantly reduced dental caries, where the difference among children from employed fathers was largest (Dargent-Paré et al. 1999). Another study evaluated the effect of an extended preventive program where the intervention group received additional preventive measures (i.e., brushing exercises, dental screening, fluoride varnish) on top of the basic program (yearly lessons and education on oral health) at primary schools. Children with an ethnic majority background showed a significant reduction in dental caries, where the group of Grammar students showed the greatest absolute difference (Heinrich-Weltzien et al. 2007). The third study evaluated the effect of biweekly supervised mouth rinsing at school with a fluoride solution, compared to children receiving no intervention. Only children from the least deprived areas showed a statistically significant decrease in dental caries (Levin et al. 2009). The last study evaluated the effect of oral health education provided by schoolteachers at primary schools, and the authors of this study calculated the incidence rate ratio in both socioeconomic groups. The educational program showed a significant protective effect on the development of dental caries in children with high SES but not with low SES (Qadri et al. 2018).

#### Interventions that may have no impact on inequalities

Five studies showed results that did not have a differential impact on oral health inequalities (Winter et al. 1989; Freeman et al. 2001; Ellwood et al. 2004; Drosen et al. 2010; Winter et al. 2018) (Table 2 and Appendix Table 2). One study evaluated the effect of an extended preventive program, including the basic program and additional brushing exercises, fluoride varnish, and educational games, compared to a basic program that included the yearly instruction on caries prevention and supervised toothbrushing and dental flossing. Children from both ethnic majority and ethnic minority groups did not show a statistically significant difference in dental caries, although a small reduction in dental caries was observed among children with an ethnic minority background (Drosen et al. 2010). The second study evaluated the effect on dental caries in 2 intervention groups that were provided with dental health literature, toothbrush, and free toothpaste containing either 440 or 1,450 ppm fluoride. Of the intervention group that received 1,450 ppm fluoride toothpaste, children from the nondeprived and deprived areas showed a similar absolute reduction in dental caries. Children from the intervention group receiving 440 ppm fluoride and belonging to the most deprived areas showed a nonsignificant reduction in dental caries, whereas children from the nondeprived areas showed a nonsignificant increase in dental caries (Ellwood et al. 2004). Another study evaluated the effect of a break policy at primary schools where only the consumption of milk and fruit was allowed. In both socioeconomic groups, the intervention did not significantly change the risk of dental caries (Freeman et al. 2001). The fourth study studied the effect of the provision of free toothpaste with a high fluoride content (1,055 ppm) compared to free toothpaste with low fluoride content (550 ppm). Children with both manual and nonmanual occupational class parents did not show a significant difference in dental caries (Winter et al. 1989). The last study evaluated the effect of 5 different interventions, including several components at kindergarten and primary schools (ranging from intensive to less intensive program elements), compared to children who did receive basic dental education at primary school. Although children from low SES receiving interventions 3 and 4 (receiving few times toothbrushing exercises or instructions on toothbrushing) showed an increase in caries, no statistically significant differences in effect on dental caries were observed between interventions 1, 3, 4, and 5 (Winter et al. 2018).

### Common Characteristics of Interventions

Of the 6 studies that were indicative of a potential reduction in oral health inequalities (Evans et al. 1996; McMahon et al. 2011; MacPherson et al. 2013; Wagner and Heinrich-Weltzien 2017; Winter et al. 2018; Kidd et al. 2020), 3 evaluated (a part) of the “Childsmile” intervention (McMahon et al. 2011; MacPherson et al. 2013; Kidd et al. 2020). Five interventions included a daily exposure to fluoride via water or toothbrushing (Evans et al. 1996; McMahon et al. 2011; MacPherson et al. 2013; Winter et al. 2018; Kidd et al. 2020), 3 were performed from birth onward (Evans et al. 1996; McMahon et al. 2011; Wagner and Heinrich-Weltzien 2017), duration varied from 2 to 6 y, and the setting differed per study. All studies evaluated dental caries in the deciduous dentition and 1 additionally in permanent teeth (Winter et al. 2018). Furthermore, all programs incorporated the wider organizational or public policy level by implementing a setting where teeth are brushed at school regularly or having fluoridated water.

Of the 4 studies that were indicative of a potential widening in oral health inequalities (Dargent-Paré et al. 1999; Heinrich-Weltzien et al. 2007; Levin et al. 2009; Qadri et al. 2018), 3 had an infrequent exposure to the intervention program (Heinrich-Weltzien et al. 2007; Levin et al. 2009; Qadri et al. 2018), and for 1 study, frequency was unknown (Dargent-Paré et al. 1999). All studies were performed in primary schools and evaluated dental caries in the permanent dentition (Dargent-Paré et al. 1999; Heinrich-Weltzien et al. 2007; Levin et al. 2009; Qadri et al. 2018). Regarding the level of influence, 1 study focused at the individual and interpersonal level (Qadri et al. 2018) (i.e., having oral health education in class), and 3 others included a combination of the individual (i.e., provision of fluoride) or interpersonal (i.e., oral health education in class) and organizational level (i.e., implementing a fixed moment of mouth rinsing or toothbrushing in class) (Dargent-Paré et al. 1999; Heinrich-Weltzien et al. 2007; Levin et al. 2009).

Of the 5 studies that showed results that did not indicate a differential impact on oral health inequalities (Winter et al. 1989; Freeman et al. 2001; Ellwood et al. 2004; Drosen et al. 2010; Winter et al. 2018), 3 were performed at primary schools (Freeman et al. 2001; Drosen et al. 2010; Winter et al. 2018) and 2 at a home setting (Winter et al. 1989; Ellwood et al. 2004). The program frequency was mainly once or a few times a year (Winter et al. 1989; Ellwood et al. 2004; Drosen et al. 2010; Winter et al. 2018). The age when the intervention started and its duration varied. Two studies evaluated caries in mixed dentition (Drosen et al. 2010; Winter et al. 2018), 2 in deciduous dentition (Winter et al. 1989; Ellwood et al. 2004), and 1 in permanent dentition (Freeman et al. 2001). Also, the level of influence varied per study, with 2 studies solely focusing on the individual level (i.e., provision of toothpaste or toothbrush) (Winter et al. 1989; Ellwood et al. 2004), 1 merely on the organizational level (i.e., nutrition guidelines for school break) (Freeman et al. 2001), and 2 incorporated a mix of individual, interpersonal, and organizational level (i.e., oral health education, provision of fluoride or toothbrush, supervised toothbrushing in class, or fluoride varnish application) (Drosen et al. 2010; Winter et al. 2018) (Table 3).

**Table 3.** Common Characteristics of Interventions Sorted by Their Potential Impact on Oral Health Inequalities.

Potential Impact on Inequalities^a^	Intervention	Start Period	Duration	Setting	Outcome	Level of Influence
**↓**	Fluoridated water (Evans et al. 1996)	Birth	5 y	Community	Deciduous dentition	Public policy
	Daily supervised toothbrushing (Childsmile program) (Kidd et al. 2020)	3 y	1–3 y	Nursery school	Deciduous dentition	Interpersonal and organizational
	Daily supervised toothbrushing and free toothpaste (Childsmile program) (MacPherson et al. 2013)	3 y	2 y	Nursery school and home	Deciduous dentition	Individual, interpersonal, and organizational
	Multicomponent Childsmile program, including at least provision of toothpaste and brush, daily supervised toothbrushing, and healthy snacks at schools (McMahon et al. 2011)	Birth	3 y	Community, dental practice, home, nursery and primary schools	Deciduous dentition	Individual, interpersonal, and organizational
	Extended oral health program including few times counseling and 2 to 4 fluoride varnish applications (Wagner and Heinrich-Weltzien 2017)	Birth	5 y	Home and dental clinic	Deciduous dentition	Individual and organizational
	Multicomponent intervention including daily supervised toothbrushing in kindergarten, free toothpaste and toothpaste, and few times per year brushing exercises in class (Winter et al. 2018)	3 y	6 y	Kindergarten and primary schools	Mixed dentition	Individual, interpersonal, and organizational
**↑**	Multicomponent intervention including fluoride provision, education, and toothbrushing (Dargent-Paré et al. 1999)	3 y	8 y	Primary school	Permanent dentition	Individual, interpersonal, and organizational
	Oral health program including few times per year brushing, screening, education, and fluoride varnish (Heinrich-Weltzien et al. 2007)	2 y	10 y	Kindergarten and primary school	Permanent dentition	Individual, interpersonal, and organizational
	Biweekly supervised mouth rinsing with fluoride (Levin et al. 2009)	6 y	5 y	Primary schools	Permanent dentition	Interpersonal and organizational
	Oral health education at school through schoolteachers (Qadri et al. 2018)	8 y	1.5 y	Primary school	Permanent dentition	Individual and interpersonal
**↔**	Intensive preventive program including healthy breakfast, toothbrushing, education, and fluoride varnish once a year (Drosen et al. 2010)	6 y	4 y	Primary schools	Mixed dentition	Individual, interpersonal, and organizational
	Provision of free toothpaste, toothbrush, and dental education every 3 mo via post (Ellwood et al. 2004)	Birth	5 y	Home	Deciduous dentition	Individual
	School break policy consumption of only milk and fruit (Freeman et al. 2001)	8 y	2 y	Primary schools	Permanent dentition	Organizational
	Provision of free toothpaste and toothbrush (Winter et al. 1989)	2 y	3 y	Home	Deciduous dentition	Individual
	Multicomponent interventions ranging in intensity including supervised toothbrushing, exercises, and free toothpaste and toothpaste, provided daily or few times a year (Winter et al. 2018)	3 y	6 y	Kindergarten and primary schools	Mixed dentition	Individual, interpersonal, and organizational

a The potential impact on inequalities is reported using symbols indicating the following: ↓ if the intervention is likely to reduce inequalities, ↑ if the intervention is likely to widen inequalities, and ↔ if the intervention had no differential impact on the 2 groups.

## Discussion

### Main Results

The present scoping review identified (components of) interventions that have the potential to reduce oral health inequalities among children. Of the included studies, 6 presented intervention strategies with a potential reduction in oral health inequalities, 4 presented intervention strategies that were indicative of a widening in oral health inequalities, and 5 presented intervention strategies that were indicative of no preferential impact on oral health inequalities. Common characteristics of interventions that may reduce oral health inequalities were a high frequency of the intervention program (daily), an early onset (from birth onward), targeting at the deciduous dentition, and incorporating at least the organizational or public policy level regarding the level of influence.

### Explanation of Results

In our study, we found that the timing of the intervention, from birth onward, may play a role in the effectiveness of the intervention, especially among the most disadvantaged groups. This is supported by a previous study that concluded that targeting children at a younger age (before 24 months) could lead to greater reductions in early childhood caries (Hirsch et al. 2012). Also, another study revealed the importance of intervening during the early years of a child’s life for future oral health (Chen et al. 2007). Besides, in older children with permanent dentition, disparities might be already wider due to long-term poor lifestyle behaviors that could already be present at an early age and continued throughout the years (Harris et al. 2006; Verlinden et al. 2019; Lioret et al. 2020). This theory is also supported by the WHO, which supports a good start to life for every child as a key strategy to limit the effects of social disadvantage on health during childhood and to reduce the risk of several chronic and lifestyle diseases (Marmot et al. 2012). Furthermore, the interventions with frequent contact moments had a higher potential to reduce oral health inequalities than less intensive health programs. The idea that repeated exposure to a beneficial health behavior increases health improvement over time, and thus that the intensity and frequency of an intervention program is an important element of an intervention to succeed, has been described earlier in the intervention mapping approach (Whitlock et al. 2002). Last, our review indicates that the level of influence may be a potential factor that affects the direction of interventions on oral health inequalities. The level of influence is based on an ecologic perspective model, which is advised to use for health promotion programs and when health behaviors need to be changed (McLeroy et al. 1988; Rimer and Glanz 2005). Most of the interventions that relied on only one of the levels of influence were equally effective in both social groups, whereas intervention programs that aimed to influence multiple levels, but at least and especially the broader organizational or public policy level (such as fluoridated water or provision of fluoride at school), showed a reduction in dental caries in particular among children from the most disadvantaged groups. This is supported by a recent review of Shen et al. (2021), which reported that interventions that aim to target the whole population are more likely to reduce oral health inequalities among children. Previous literature already indicated that when interventions solely rely on the individual voluntary behavior change, so-called downstream interventions, they may increase health inequalities (Mechanic 2002; Lorenc et al. 2013). This is due to the individuals’ lack of resources to adopt preventive and healthy behaviors, such as knowledge, self-efficacy, and social network (McLaren et al. 2010). On the other hand, “upstream” interventions try to influence the social or policy environment and are often recognized as interventions that are more likely to reduce health inequalities (Lorenc et al. 2013). “Upstream” interventions address the underlying cause of health disparities, by making adaptations to the social or physical environment of individuals. Therefore, “upstream” interventions will most likely reach the whole population, and especially the most disadvantaged groups will benefit since few individual-level actions or resources are required (Whitehead 2007; McLaren et al. 2010). The WHO supports this by setting goals to limit health disparities, which underlines that all levels from the wider society and system should be involved when taking action to improve equal health to the whole population (Marmot et al. 2012).

### Strength and Limitations

A strength of this scoping review is the comprehensive literature search in multiple databases. We aimed to include all relevant articles without language or publication year limits. Furthermore, we also included nonrandomized studies in this review. Although these individual studies may be more prone to bias, by including these studies, we were able to evaluate the effect of certain policies or regulations implemented at schools or communities and thereby provide a more comprehensive overview of potential measures that could be implemented to reduce oral health inequalities in the future. On the other hand, there are also limitations to consider. A limited number of relevant studies could be included in this scoping review, as we had to exclude 63 studies because of the nonstratification of social groups. Also, of the limited studies that were included, 6 were of low quality. Therefore, the results of this scoping review should be interpreted with caution. However, it clearly identifies the need for high-quality studies when aiming to reduce dental caries across different social groups. If randomization is not possible, a proper description of the study population, including an overview of baseline characteristics, or taking measures to reduce loss to follow-up, could ensure that the internal validity of individual studies will not be highly affected. We only included studies from European countries, although other studies have been performed that evaluated the effect of interventions across different social groups in other continents. Nevertheless, we decided to only include European studies since these will be more homogeneous regarding fluoridated water laws, health care and school systems, and the cultural and socioeconomic backgrounds of the population. Consequently, the results of this review are applicable for and generalized to a European population setting. Another limitation is that each study determined socially disadvantaged groups differently. Also, the determination and comparison of the lowest and highest groups might differ per study. For example, when the most extreme social groups are compared (e.g., lowest 10% versus highest 10%), a much larger difference will be revealed than comparing the lowest 50% versus the highest 50% of a total population, and this might influence the conclusion regarding the potential impact on oral health inequalities per study. Moreover, in our study, we evaluate the potential impact on oral health inequalities using the (significance of) effect estimates per social group, by only including studies that reported the effect of the intervention in groups of children with different social backgrounds separately. It should be noted that other methods exist to evaluate the impact on oral health inequalities potentially leading to a divergent conclusion (King et al. 2012). Furthermore, our scoping review mainly relies on the statistical significance of the individual study results to evaluate the differential impact of the intervention in each social group, which is highly influenced by the sample sizes of each study group (Appendix Table 2). Since most studies that were included in this review were initially not designed to study the effect of the intervention in different social groups of children separately, the sample size of each social group might be underpowered. Therefore, we cannot exclude the possibility that interventions with an effect size that clearly showed an absolute mean difference would have shown statistically significant results and a potential impact on oral health inequalities if it would have larger power.

## Conclusions

The need for interventions to reduce oral health inequalities has been previously recognized. This review shows that oral health interventions may have differential effects across children from birth to 12 y from different social groups. Interventions that contain early approaches, with a high frequency, and influence multiple levels, including the broader organizational or public policy level, are most promising to reduce oral health inequalities among children. Still, the causes of inequalities in oral health remain interrelated and complex. The number of high-quality studies is very limited, which subsequently limits the conclusion of this review. In order to come up with thoroughly based conclusions, we first need more properly designed intervention studies in the field of preventive dentistry. In addition, we suggest to evaluate the effectiveness of an oral health intervention always in socioeconomic or ethnic groups separately, to better understand the effect of the intervention on oral health inequalities.

## Author Contributions

A.W. Van Meijeren-van Lunteren, contributed to conception, design, data acquisition, analysis, and interpretation, drafted and critically revised the manuscript; Y. Yue, contributed to data acquisition, critically revised the manuscript; H. Raat, contributed to design, critically revised the manuscript; E.B. Wolvius, contributed to conception, critically revised the manuscript; L. Kragt, contributed to conception, design, and data interpretation, critically revised the manuscript. All authors gave final approval and agree to be accountable for all aspects of the work.

## Supplemental Material

sj-docx-1-jct-10.1177_23800844221109116 – Supplemental material for Caries Preventive Interventions and Oral Health Inequalities: A Scoping Review Click here for additional data file. Supplemental material, sj-docx-1-jct-10.1177_23800844221109116 for Caries Preventive Interventions and Oral Health Inequalities: A Scoping Review by A.W. van Meijeren-van Lunteren, Y. You, H. Raat, E.B. Wolvius and L. Kragt in JDR Clinical & Translational Research
