# Supplementary material for: Caries Preventive Interventions and Oral Health Inequalities: A Scoping Review
Source: JDR Clin Trans Res. 2022 Jul 31;8(4):311–25. doi: 10.1177/23800844221109116 (PMC10504820; doi:10.1177/23800844221109116)
Supplement: sj-docx-1-jct-10.1177_23800844221109116 – Supplemental material for Caries Preventive Interventions and Oral Health Inequalities: A Scoping Review [file sj-docx-1-jct-10.1177_23800844221109116.docx]

***Supplementary material***

**Article title:** Caries Preventive Interventions and Oral Health Inequalities-A Scoping Review
**Authors:** Agatha W. van Meijeren - van Lunteren, MSc; Yueyue You, MSc; Hein Raat, MD, PhD, MBA; Eppo B. Wolvius; and Lea Kragt, PhD
**Content of this file:** This electronic supplementary material contains tables with the results of several additional content or supplemental tables which support the results of the main manuscript.

# Search strategy

Search performed 22 November 2019 and an update on 15 March 2021

**Embase.com**

('dental caries'/de OR 'caries assessment'/exp OR 'oral hygiene index'/de OR 'dental procedure'/de OR 'dental health'/de OR (caries OR cariogenesis OR ((carious OR decay) NEAR/3 (tooth OR teeth OR lesion*)) OR dental-procedure OR dental-health OR oral-health OR DMF-ind* OR DMFS-ind* OR DMFT-ind* OR white-spot* OR oral-hygiene-ind*):ab,ti,kw) **AND** ('preventive medicine'/de OR 'preventive dentistry'/de OR 'prevention and control'/de OR 'prevention'/de OR 'prevention':lnk OR 'prophylaxis'/de OR 'prevention study'/de OR 'dental health education'/de OR 'primary prevention'/de OR 'dental prevention'/exp OR 'health program'/exp OR 'health education'/exp OR 'public health'/de OR 'public health service'/de OR 'community program'/de OR 'education program'/de OR 'public health campaign'/de OR 'school dentistry'/de OR (((health OR dental*) NEAR/3 (educat* OR promot*)) OR (health* NEAR/3 (program* OR campaign*)) OR (public* NEAR/3 health*) OR prevent* OR promot* OR prophylaxis* OR ((communit* OR educat*) NEAR/3 program*)):ab,ti,kw) **AND** ('intervention study'/de OR 'community trial'/de OR 'family study'/de OR 'controlled clinical trial'/exp OR 'controlled study'/exp OR 'population research'/de OR 'evaluation research'/de OR 'comparative study'/exp OR 'quasi experimental study'/exp OR 'early childhood intervention'/de OR 'outcome assessment'/de OR 'program evaluation'/exp OR (intervention* OR ((communit* OR public) NEXT/1 (trial* OR health)) OR program* OR ((effect* OR research*) NEAR/3 evaluation*) OR trial* OR (population NEXT/1 research) OR ((compar* OR prevent* OR quasi-experiment* OR quasiexperiment* OR controlled) NEAR/3 stud*) OR (assess* NEAR/3 outcome*)):ab,ti,kw) **AND** ('socioeconomics'/de OR 'household economic status'/de OR 'social status'/exp OR 'income group'/exp OR 'educational status'/exp OR 'ethnic or racial aspects'/exp OR 'ethnic group'/exp OR 'ancestry group'/exp OR 'migration'/exp OR 'minority group'/de OR (socioeconomic* OR socio-economic* OR socio-demographic* OR sociodemographic* OR ((household* OR famil*) NEAR/3 (economic-status)) OR poverty OR income* OR (education* NEAR/3 (status OR level*)) OR literacy OR ((read*) NEAR/3 (abilit* OR skill*)) OR ethnic* OR ancestr* OR race* OR racial* OR multiracial* OR migrant* OR immigrant* OR refugee* OR asylum-seek* OR non-native* OR ((neighborhood* OR neighbourhood* OR social* OR area*) NEAR/3 (depriv*)) OR ((occupation* OR job* OR work*) NEAR/3 (class* OR status*)) OR social-status OR ((social*) NEAR/3 (disadvantage*)) OR ((minorit*) NEAR/3 (group*))):ab,ti,kw) AND (child/exp OR 'child behavior'/de OR 'early childhood intervention'/de OR pediatrics/exp OR childhood/exp OR 'child nutrition'/de OR 'infant nutrition'/exp OR 'child health'/de OR 'child health care'/exp OR 'child care'/exp OR 'childhood disease'/exp OR (infan* OR baby OR babies OR child* OR kid OR kids OR toddler* OR teen* OR boy* OR girl* OR minors OR underag* OR (under NEXT/1 (age* OR aging OR ageing)) OR juvenil* OR youth* OR kindergar* OR pediatric* OR paediatric* OR school* OR preschool* OR suckling*):ab,ti,kw) NOT ([Conference Abstract]/lim AND [1800-2017]/py)

**Medline (Ovid)**

(exp Dental Caries/ OR Dental Caries Susceptibility/ OR Oral Hygiene Index/ OR DMF Index/ OR Dental Care/ OR Dental Care for Children/ OR Oral Health/ OR (caries OR cariogenesis OR ((carious OR decay) ADJ3 (tooth OR teeth OR lesion*)) OR dental-procedure OR dental-health OR oral-health OR DMF-ind* OR DMFS-ind* OR DMFT-ind* OR white-spot* OR oral-hygiene-ind*).ab,ti,kw.) **AND** (Preventive Medicine/ OR Preventive Dentistry/ OR Dental Prophylaxis/ OR Preventive Health Services/ OR (prevention and control).fs. OR Primary Prevention/ OR National Health Programs/ OR exp Health Education/ OR Public Health/ OR Public Health Practice/ OR Public Health Dentistry/ OR Health Promotion/ OR Healthy People Programs/ OR Dental Care/ OR Dental Care for Children/ OR School Dentistry/ OR (((health OR dental*) ADJ3 (educat* OR promot*)) OR (health* ADJ3 (program* OR campaign*)) OR (public* ADJ3 health*) OR prevent* OR promot* OR prophylaxis* OR ((communit* OR educat*) ADJ3 program*)).ab,ti,kw.) **AND** (exp Clinical Trial/ OR Comparative Study/ OR Early Medical Intervention/ OR "Outcome Assessment (Health Care)"/ OR Program Evaluation/ OR (intervention* OR ((communit* OR public) ADJ (trial* OR health)) OR program* OR ((effect* OR research*) ADJ3 evaluation*) OR trial* OR (population ADJ research) OR ((compar* OR prevent* OR quasi-experiment* OR quasiexperiment* OR controlled) ADJ3 stud*) OR (assess* ADJ3 outcome*)).ab,ti,kw.) **AND** (exp Socioeconomic Factors/ OR exp Educational Status/ OR Race Factors/ OR exp Population Groups/ OR exp Human Migration/ OR Refugees/ OR Minority Groups/ OR (socioeconomic* OR socio-economic* OR socio-demographic* OR sociodemographic* OR ((household* OR famil*) ADJ3 (economic-status)) OR poverty OR income* OR (education* ADJ3 (status OR level*)) OR literacy OR ((read*) ADJ3 (abilit* OR skill*)) OR ethnic* OR ancestr* OR race* OR racial* OR multiracial* OR migrant* OR immigrant* OR refugee* OR asylum-seek* OR non-native* OR ((neighborhood* OR neighbourhood* OR social* OR area*) ADJ3 (depriv*)) OR ((occupation* OR job* OR work*) ADJ3 (class* OR status*)) OR social-status OR ((social*) ADJ3 (disadvantage*)) OR ((minorit*) ADJ3 (group*))).ab,ti,kw.) AND (exp Child/ OR exp Infant/ OR exp Child Behavior/ OR exp Pediatrics/ OR Child Nutrition Sciences/ OR Infant nutritional physiological phenomena/ OR exp Child Health Services/ OR exp Child Care/ OR (infan* OR baby OR babies OR child* OR kid OR kids OR toddler* OR teen* OR boy* OR girl* OR minors OR underag* OR (under ADJ (age* OR aging OR ageing)) OR juvenil* OR youth* OR kindergar* OR pediatric* OR paediatric* OR school* OR preschool* OR suckling*).ab,ti,kw.) NOT (news OR congres* OR abstract* OR book* OR chapter* OR dissertation abstract*).pt.

**Web of Science Core Collection**

TS=(((caries OR cariogenesis OR ((carious OR decay) NEAR/2 (tooth OR teeth OR lesion*)) OR dental-procedure OR dental-health OR oral-health OR DMF-ind* OR DMFS-ind* OR DMFT-ind* OR white-spot* OR oral-hygiene-ind*)) **AND** ((((health OR dental*) NEAR/2 (educat* OR promot*)) OR (health* NEAR/2 (program* OR campaign*)) OR (public* NEAR/2 health*) OR prevent* OR promot* OR prophylaxis* OR ((communit* OR educat*) NEAR/2 program*))) **AND** ((intervention* OR ((communit* OR public) NEAR/1 (trial* OR health)) OR program* OR ((effect* OR research*) NEAR/2 evaluation*) OR trial* OR (population NEAR/1 research) OR ((compar* OR prevent* OR quasi-experiment* OR quasiexperiment* OR controlled) NEAR/2 stud*) OR (assess* NEAR/2 outcome*))) **AND** ((socioeconomic* OR socio-economic* OR socio-demographic* OR sociodemographic* OR ((household* OR famil*) NEAR/2 (economic-status)) OR poverty OR income* OR (education* NEAR/2 (status OR level*)) OR literacy OR ((read*) NEAR/2 (abilit* OR skill*)) OR ethnic* OR ancestr* OR race* OR racial* OR multiracial* OR migrant* OR immigrant* OR refugee* OR asylum-seek* OR non-native* OR ((neighborhood* OR neighbourhood* OR social* OR area*) NEAR/2 (depriv*)) OR ((occupation* OR job* OR work*) NEAR/2 (class* OR status*)) OR social-status OR ((social*) NEAR/2 (disadvantage*)) OR ((minorit*) NEAR/2 (group*)))) AND ((infan* OR baby OR babies OR child* OR kid OR kids OR toddler* OR teen* OR boy* OR girl* OR minors OR underag* OR (under NEAR/1 (age* OR aging OR ageing)) OR juvenil* OR youth* OR kindergar* OR pediatric* OR paediatric* OR school* OR preschool* OR suckling*)))

**Cochrane Central Register of Controlled Trials**

((caries OR cariogenesis OR ((carious OR decay) NEAR/3 (tooth OR teeth OR lesion*)) OR dental-procedure OR dental-health OR oral-health OR DMF-ind* OR DMFS-ind* OR DMFT-ind* OR white-spot* OR oral-hygiene-ind*):ab,ti,kw) **AND** ((((health OR dental*) NEAR/3 (educat* OR promot*)) OR (health* NEAR/3 (program* OR campaign*)) OR (public* NEAR/3 health*) OR prevent* OR promot* OR prophylaxis* OR ((communit* OR educat*) NEAR/3 program*)):ab,ti,kw) **AND** ((intervention* OR ((communit* OR public) NEXT/1 (trial* OR health)) OR program* OR ((effect* OR research*) NEAR/3 evaluation*) OR trial* OR (population NEXT/1 research) OR ((compar* OR prevent* OR quasi-experiment* OR quasiexperiment* OR controlled) NEAR/3 stud*) OR (assess* NEAR/3 outcome*)):ab,ti,kw) **AND** ((socioeconomic* OR socio-economic* OR socio-demographic* OR sociodemographic* OR ((household* OR famil*) NEAR/3 (economic-status)) OR poverty OR income* OR (education* NEAR/3 (status OR level*)) OR literacy OR ((read*) NEAR/3 (abilit* OR skill*)) OR ethnic* OR ancestr* OR race* OR racial* OR multiracial* OR migrant* OR immigrant* OR refugee* OR asylum-seek* OR non-native* OR ((neighborhood* OR neighbourhood* OR social* OR area*) NEAR/3 (depriv*)) OR ((occupation* OR job* OR work*) NEAR/3 (class* OR status*)) OR social-status OR ((social*) NEAR/3 (disadvantage*)) OR ((minorit*) NEAR/3 (group*))):ab,ti,kw) AND ((infan* OR baby OR babies OR child* OR kid OR kids OR toddler* OR teen* OR boy* OR girl* OR minors OR underag* OR (under NEXT/1 (age* OR aging OR ageing)) OR juvenil* OR youth* OR kindergar* OR pediatric* OR paediatric* OR school* OR preschool* OR suckling*):ab,ti,kw)

Appendix Table 1 - Extensive quality appraisal of the studies included in the review

| Reference | Selection Bias | Study design | Confounders | Blinding | Data collection methods | Withdrawals and drop outs | Global rating |
| --- | --- | --- | --- | --- | --- | --- | --- |
| Dargent-Paré et al. (1999) | Strong | Weak | Strong | Weak | Strong | Not Applicable | Weak |
| Drosen et al. (2010) | Moderate | Moderate | Weak | Weak | Moderate | Weak | Weak |
| Ellwood et al. (2004) | Strong | Strong | Weak | Moderate | Strong | Weak | Weak |
| Evans et al. (1996) | Strong | Moderate | Strong | Weak | Strong | Moderate | Moderate |
| Freeman et al. (2001) | Strong | Moderate | Strong | Moderate | Strong | Weak | Moderate |
| Heinrich-Weltzien et al. (2007) | Moderate | Weak | Weak | Weak | Moderate | Not Applicable | Weak |
| Kidd et al. (2020) | Strong | Moderate | Strong | Moderate | Strong | Strong | Strong |
| Levin et al. (2009) | Strong | Moderate | Strong | Moderate | Strong | Weak | Moderate |
| MacPherson et al. (2013) | Moderate | Weak | Weak | Moderate | Strong | Not Applicable | Weak |
| McMahon et al. (2011) | Moderate | Weak | Weak | Moderate | Strong | Not Applicable | Weak |
| Qadri et al. (2018) | Moderate | Strong | Strong | Moderate | Strong | Strong | Strong |
| Wagner and Heinrich-Weltzien (2017) | Strong | Moderate | Strong | Moderate | Strong | Weak | Moderate |
| Winter et al. (1989) | Moderate | Strong | Strong | Strong | Strong | Moderate | Strong |
| Winter et al. (2018) | Moderate | Strong | Moderate | Moderate | Strong | Weak | Moderate |

Global rating: Strong, no weak and at least 4 strong ratings; Moderate, less than 4 strong and 1 weak rating; Weak, 2 or more weak ratings

**Appendix Table 2 – Extended table on the effectiveness per intervention, stratified per social group**

| Study ref | Outcome | Socially disadvantaged group | Intervention | | | Control | | | MD  (95% CI) | RR (95% CI) | Potential impact on oral inequalities^a^ |
| --- | --- | --- | --- | --- | --- | --- | --- | --- | --- | --- | --- |
|  |  |  | n | Mean (SD) | Proportion (%) | n | Mean (SD) | Proportion (%) |  |  |  |
| Dargent-Paré et al. (1999) | DMFT | Employed father  Unemployed father | 2242  122 | 1.95 (2.09)  2,39 (2,43) |  | 1608  112 | 3.37 (2.49)  3,52 (2.51) |  | **-1.42 (-1.57, -1.27)**  **-1.13 (-1.76, -0.50)** |  | ↑ |
| Drosen et al. (2010) | dmft/DMFT | Ethnic majority  Ethnic minority | 64  63 | 2.1 (2.3)  1.7 (2.1) |  | 69  37 | 1.9 (2.3)  2.2 (2.2) |  | 0.30 (-0.48, 1.08)  -0.50 (-1.38, 0.38) |  | *↔* |
| Ellwood et al. (2004) | dmft | Quartile 1 (least deprived)  Quartile 4 (most deprived)  Quartile 1 (least deprived)  Quartile 4 (most deprived) | **G1^b^**  280  264  **G2^b^**  251  267 | 1.4 (2.5)  2.7 (3.0)  2.2 (3.0)  2.9 (3.6) | 112 (40%)  161 (61%)  129 (51%)  157 (59%) | 328  335  328  335 | 1.9 (2.9)  3.2 (3.4)  1.9 (2.9)  3.2 (3.4) | 145 (44%)  228 (68%)  145 (44%)  228 (68%) | **-0.50 (-0.93 -0.07)**  -0.50 (-1.01, 0.01)  0.30 (-0.19, 0.79)  -0.30 (-0.86, 0.26) |  | ↔  ↔ |
| Evans et al. (1996) | dmft | Social class I + II (High)  Social class IV+V (Low) | 92  36 | 0.59 (1.37)  1.17 (2.73) |  | 79  34 | 1.46 (2.61)  2.74 (3.05) |  | **-0.87 (-1.51, -0.23)**  **-1.57 (-2.93, -0.21)** |  | ↓ |
| Freeman et al. (2001) | DMFT | High SES  Low SES | 58  41 |  | 44 (76%)  34 (83%) | 55  58 |  | 32 (58%)  47 (81%) |  | 1.30 (1.00, 1.70)  1.02 (0.85, 1.23) | ↔ |
| Heinrich-Weltzien et al. (2007) | DMFT | German students at Grammar schools  German students at secondary modern schools  Turkish students at secondary modern schools | 203  190  112 | 0.3 (0.9)  1.8 (2.5)  1.8 (1.8) |  | 171  90  91 | 1.8 (2.3)  2.5 (2.5)  2.3 (2.5) |  | **-1.50 (-1.87, -1.13)**  **-0.70 (-1.33, -0.07)**  -0.50 (-1.11, 0.11) |  | ↑ |
| Kidd et al. (2020) | Caries experience observed | SIMD 5 (least deprived)  SIMD 1 (most deprived)  SIMD 5 (least deprived)  SIMD 1 (most deprived)  SIMD 5 (least deprived)  SIMD 1 (most deprived)  SIMD 5 (least deprived)  SIMD 1 (most deprived) | **G1^b^**  522  1279  **G2^b^**  2052  3473  **G3^b^**  3664  5410  **G4^b^**  1228  941 |  | 97 (18.6%)  585 (45.7%)  341 (16.6%)  1565 (45.1%)  590 (16.1%)  2449 (45.3%)  152 (12.4%)  358 (38.0%) | 1559  674  1559  674  1559  674  1559  674 |  | 242 (15.5%)  353 (52.4%)  242 (15.5%)  353 (52.4%)  242 (15.5%)  353 (52.4%)  242 (15.5%)  353 (52.4%) |  | 1.20 (0.97, 1.48)  **0.87 (0.80, 0.96)**  1.07 (0.92, 1.25)  **0.86 (0.79, 0.93)**  1.04 (0.90, 1.19)  **0.86 (0.80, 0.93)**  **0.80 (0.66, 0.96)**  **0.73 (0.65, 0.81)** | ↓  ↓  ↓  ↓ |
| Levin et al. (2009) | D3MFT | Depcat 1 (least deprived)  Depcat 7 (most deprived) | 21  32 | 0,33 (0.77)  2,16 (1.80) | 4 (19%)  22 (69%) | 59  30 | 0,83 (1.07)  2,47 (2.73) | 24 (41%)  20 (67%) | **-0.50 (-0.93, -0.07)**  -0.31 (-1.47, 0.85) |  | ↑ |
| MacPherson et al. (2013) | d3mft | Depcat 1-2 (least deprived)  Depcat 6-7 (most deprived) |  | 1,10 (2,29)  2,77 (3,59) |  |  | 1.52 (2.63)  4,48 (4,12) |  | **-0.43 (-0.60, -0.25)**  **-1.71 (-1.93, -1.49)** |  | ↓ |
| McMahon et al. (2011) | d3mft | SIMD 5 (least deprived)  SIMD 1 (most deprived) | 172  1384 | 0,1 (0,4)  0,5 (1,2) | 9 (5%)  333 (24%) | 356  655 | 0,3 (1,2)  1,5 (3,0) | 46 (13%)  216 (33%) | **-0.20 (-0.34. -0.06)**  **-1.00 (-1.24, -0.76)** |  | ↓ |
| Qadri et al. (2018) | ∆DMFT | High SES  Low SES | 84  97 |  |  | 129  68 |  |  |  | **0.09**  1.43 | ↑ |
| Wagner and Heinrich-Weltzien (2017) | d1-4mfs | High SES  Low SES | 52  19 | 0,1 (0,4)  1,0 (2,5) |  | 30  11 | 2,7 (5,6)  12,5 (11,9) |  | **-2.60 (-4.61, -0.59)**  **-11.50 (-18.62, -4.38)** |  | **↓** |
| Winter et al. (1989) | dmfs | Social class I-IIIN (non-manual occupation class)  Social class IIIM-V (manual occupation class) | 183  224 | 1,63 (4.04)  3,06 (4.90) | 71 (39%)  125 (56%) | 161  212 | 1,68 (4.01)  2,46 (4.90) | 50 (31%)  102 (48%) | -0.05 (-0.90, 0.80)  0.60 (-0.32, 1.52) |  | *↔* |
| Winter et al. (2018) | ∆dmft/DMFT | High SES  Low SES  High SES  Low SES  High SES  Low SES  High SES  Low SES  High SES  Low SES | **G1^b^**  35  23  **G2^b^**  32  21  **G3^b^**  59  65  **G4^b^**  46  60  **G5^b^**  71  123 | 0.71 (1.25)  0.78 (1.20)  1.05 (2.04)  0.50 (0.71)  0.88 (1.16)  1.16 (1.58)  0.73 (1.09)  1.25 (1.46)  0.92 (1.36)  0.89 (1.10) |  | **G6**  127  143  **G6**  127  143  **G6**  127  143  **G6**  127  143  **G6**  127  143 | 0.96 (1.23)  0.88 (1.24)  0.96 (1.23)  0.88 (1.24)  0.96 (1.23)  0.88 (1.24)  0.96 (1.23)  0.88 (1.24)  0.96 (1.23)  0.88 (1.24) |  | -0.25 (-0.72, 0.22)  -0.10 (-0.63, 0.43)  0.09 (-0.65, 0.83)  **-0.38 (-0.75, -0.01)**  -0.08 (-0.45, 0.29)  0.28 (-0.15, 0.71)  -0.23 (-0.61, 0.15)  0.37 (-0.05, 0.79)  -0.04 (-0.42, 0.34)  0.01 (-0.27, 0.29) |  | *↔*  **↓**  *↔*  *↔*  *↔* |

Bold font indicates statistical significance (p<0.05)

CI, confidence interval; dmft, decayed missing filled teeth in primary dentition; DMFT, decayed missing and filled teeth in permanent dentition; dmfs, decayed

missing and filled surfaces in primary dentition; DMFS, decayed missing and filled surfaces in permanent dentition; d3mft, modification of dmft that assesses caries

only if the decay affects the dentin layer; D3MFT, modification of DMFT that assesses caries only if the decay affects the dentin layer; d1–4mfs, modification of

dmfs that assesses caries reaching both enamel and dentin; d3–4mfs, modification of dmfs that assesses caries only if the decay affects the outer and inner dentin

layers; MD, mean difference; RR, risk ratio; SES, socioeconomic status; Δ, increment.

^a^ The potential impact on inequalities is reported using symbols indicating the following: ↓ if the intervention is likely to reduce inequalities; ↑ if the intervention is likely to widen inequalities; and ↔ if the intervention had no differential impact on the 2 groups
**^b^** The groups refer to different intervention elements as indicated in Table 1
